# T-cell epitope-based vaccine prediction against *Aspergillus fumigatus*: a harmful causative agent of aspergillosis

**DOI:** 10.1186/s43141-022-00364-x

**Published:** 2022-05-16

**Authors:** Darakshan Jabin, Ajay Kumar

**Affiliations:** Department of Biotechnology, Faculty of Engineering and Technology, Rama University, G.T. Road, Kanpur, 209217 India

**Keywords:** Aspergillosis, Immunoinformatics, Epitope, Molecular docking, MD simulation

## Abstract

**Background:**

Among the most common causes of invasive aspergillosis and acute bronchopulmonary aspergillosis is *Aspergillus fumigatus*. Transmission with *A. fumigatus* produces aggressive aspergillosis in allogeneic haematopoietic stem cell transplant recipients, HIV patients, and cancer patients. Asthmatics and cystic fibrosis patients are allergic to *A. fumigatus*. MHC class-II binding epitopes can initiate immunogenic responses in patients. In this study, we deployed immunoinformatic study to reveal epitopes from fungal proteins.

**Results:**

In modern research, we found multiple epitopes ITLKLLHRYSYKLAG, KLVLRAFPNHFRGDS, RYSYKLAGVNQVDVV, GKSFELNQAARAVTQ, and LHRYSYKLAGVNQVD from crucial proteins of *A. fumigatus* 5,8-linoleate diol synthase (ACO55067.2) and ChainB-chitinase A1 (2XVN_B). RYSYKLAGVNQVDVV, GKSFELNQAARAVTQ, and LHRYSYKLAGVNQVD epitopes interact with HLA-DRB01_0101, while ITLKLLHRYSYKLAG and KLVLRAFPNHFRGDS epitopes interact with HLA-DRB01_1501. Molecular docking analysis reveals atomic contact energy (ACE) value for these five epitopes shown below −5 Kcal/mol in docked state.

**Conclusions:**

The invasive aspergillosis and acute bronchopulmonary aspergillosis are caused by harmful fungal pathogen *Aspergillus fumigatus*. Our modern immunoinformatic research shows ITLKLLHRYSYKLAG, KLVLRAFPNHFRGDS, RYSYKLAGVNQVDVV, GKSFELNQAARAVTQ, and LHRYSYKLAGVNQVD epitopes could bind to MHC-II HLA allelic determinants and can initiate immunogenic response in patients affected by *Aspergillus fumigatus*.

## Background

Immunizing the immunosuppressed population vulnerable to opportunistic infections like aspergillosis may appear challenging; however, that could be considered as a first step and as the least immunosuppressed, most worthy prospects, including such granulomatous patients, living donor applicants before graft, leukaemic after effective initial treatment, solid tumour patients at diagnostic test, and healthcare workers with aspergillosis [1]. The far more frequent microbe fungal diseases are *Aspergillus* spp. Among the most common causes of invasive aspergillosis and acute bronchopulmonary aspergillosis is *Aspergillus fumigatus* [2]. Transmission with *A. fumigatus* produces aggressive aspergillosis in allogeneic hematopoietic stem cell transplant recipients, HIV patients, and cancer patients. Asthmatics and cystic fibrosis patients are allergic to *A. fumigatus* [3, 4]. In individuals with atopic asthma or cystic fibrosis, allergy is caused by a hypersensitivity response to *Aspergillus* allergens. When compared to other fungal allergens, diseases related with *A. fumigatus* allergens are on the rise, and it also complicates life-threatening infections in immunocompromised individuals such as cancer patients, HIV patients, and organ transplant recipients [5].

**Fig. 1:**
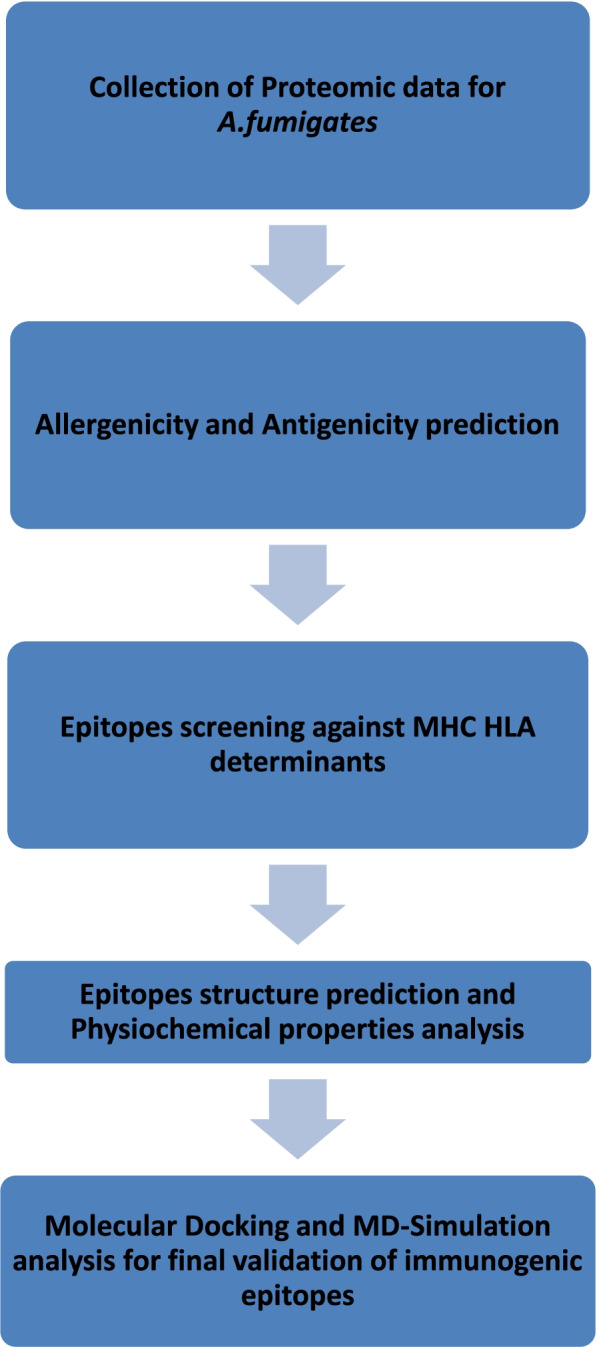
Flow chart of epitope-based vaccine prediction against *A. fumigatus*

Only a few drugs (such as voriconazole and amphotericin B) are now available to treat this invasive condition, and even these have restrictions owing to potential risks, and so, these drugs hold longer duration of treatment with side effects [6], so we tried to explore more best possible options of epitopes by deploying approaches of immunoinformatics. In current study, we targeted variety of proteins from *A. fumigatus* to screen out immunogenic T-cell epitopes against *A. fumigatus* fungi. In Fig. 1, detailed stepwise outline of epitope-based vaccine prediction strategies is provided.

## Methods

### Screening of epitopes from proteome of *A. fumigatus*

Protein database like NCBI GenBank, EMBL, and DDBJ was deployed for checking proteins of *A. fumigatus* with various accession numbers/GenBank ID. Proteins of *A. fumigatus* fungi under consideration were enlisted in Table 1.

**Table 1:** Proteins of *A. fumigatus* with accession number

S. no.	Protein name (no. of amino acids)	GenBank accession no.
1	Beta-tubulin (62 aa^a^)	AOH95126.1
2	r-ASP-4 (286 aa)	CAA04959.1
3	ChainB-chitinase A1 (309 aa^a^)	2XVN_B
4	1,4-glucan branching enzyme (56 aa^a^)	AAY83208.1
5	5,8-linoleate diol synthase (1079 aa^a^)	ACO55067.2

^a^*aa* amino acids

### Allergenicity and antigenicity prediction of proteins

AllergenFP tool [7] was deployed for prediction allergenicity on the basis of Tanimoto similarity index; also, VaxiJen ver.2.0 tool [8] was used for prediction of antigenicity of epitopes of proteins after successful epitopes screening.

### Epitope screening from proteins of *A. fumigatus*

Epitopes were screened by using NetMHCIIpan ver.3.2 server [9, 10], which screens epitopes from proteins of *A. fumigatus* via ANN algorithms. Also, affinity scores assisted in predicting binding of these epitopes to MHC-II allelic determinants. MHC-II HLA determinants were screened by using IEDB database.

### Physiochemical properties analysis

All properties of screened epitopes were determined by using ExPASy tools like ProtParam, which assisted us in finding isoelectric point (pI), GRAVY score, instability score, half-life and molecular weight, etc. [11].

### Epitope structure prediction

Many latest tools like I-TASSER [12], SWISS-PROT [13], PEP-FOLD ver.3.5 [14], and Phyre2.0 [15] tools were used for 3D structure prediction of proteins.

### Molecular docking

Molecular docking by using latest tools assisted us in finding binding scores, binding pocket, and H-bonds between epitopes and MHC-II HLA determinants. The latest tool PatchDock [16] free server is easily accessible and deployed here for docking analysis. For analysing, docked complex visualization tools like Chimera tool and PyMOL tool were used. Docked complex structural validation by using ProSA [17] and MolProbity [18] was conducted. These tools assist in determining Z-score and Ramachandran plot for protein complexes.

### Molecular dynamic and simulation

Docked complexes were used to analyse undersimulation by deploying GROMACS tool [19], which assisted us in determining stability of complex by notifying RMSD and RMSF plots. We employed an OPLS-AA force field for MD analysis, which was defined by computing the structural energy of biological and biochemical systems for 100 ns.

## Results

### Protein selection and allergenicity analysis

Core proteins of *A. fumigatus* were downloaded in fast format from GenBank-NCBI database and subjected to allergenicity analysis by using AllergenFP tool. This server produced TSI (Tanimoto similarity index) for defining resemblance of given protein sequence to reveal allergen or non-allergen nature of the given proteins (Table 2). Non-allergenic proteins were selected and further used to identify epitopes from them.

**Table 2:** Allergenicity of core proteins of *A. fumigatus* with Tanimoto similarity index

S. no.	Protein name	Tanimoto similarity index (Allergenicity)
1	Beta-tubulin	0.79 (non-allergen)
2	r-ASP-4	1.0 (allergen)
3	ChainB-chitinase A1	0.8 (non-allergen)
4	1,4-glucan branching enzyme	0.74 (non-allergen)
5	5,8-linoleate diol synthase	0.83 (non-allergen)

### Epitopes screening and antigenicity analysis

NetMHCIIpan 3.2 server was used to find epitopes of *A. fumigatus* core proteins that can bind to MHC-II HLA-DRB molecules. Considered HLA-DRB proteins were HLA-DRB01_0101, HLA-DRB01_1501, and HLA-DRB01_1101 that were used against each FASTA sequence provided to this server which is based on ANN algorithm. This server generates 1-log50K score, binding affinity in nm, and ranks. Threshold of rank under 1.5 was considered for selection of epitopes, to reveal perfect epitopes that are able to bind MHC-II allelic determinants. HLA-DRB01_0101 shows maximum binding with a total of 27 epitopes (Table 3), HLA-DRB01_1501 shows maximum binding with total of 12 epitopes (Table 4), and HLA-DRB01_1101 shows maximum binding with total of 11 epitopes (Table 5), each of 15 amino residues in length.

**Table 3:** NetMHCIIpan 3.2 scores for HLA-DRB01_0101

S. no.	Protein GenBank_ID	Epitope	DRB1_0101		
			1-log50k	nM	Rank
1	pdb_2XVN_B	VPERKFYLSAAPQCI	0.793	9.41	0.4
2	pdb_2XVN_B	PERKFYLSAAPQCII	0.824	6.74	0.09
3	pdb_2XVN_B	ERKFYLSAAPQCIIP	0.827	6.52	0.07
4	pdb_2XVN_B	RKFYLSAAPQCIIPD	0.821	6.94	0.1
5	pdb_2XVN_B	KFYLSAAPQCIIPDA	0.787	10.07	0.5
6	ACO55067.2	LHVPTVFRSIEALGI	0.796	9.12	0.4
7	ACO55067.2	HVPTVFRSIEALGIQ	0.821	6.91	0.1
8	ACO55067.2	VPTVFRSIEALGIQQ	0.828	6.41	0.06
9	ACO55067.2	PTVFRSIEALGIQQA	0.834	6.05	0.05
10	ACO55067.2	TVFRSIEALGIQQAR	0.833	6.11	0.05
11	ACO55067.2	VFRSIEALGIQQARS	0.816	7.3	0.15
12	ACO55067.2	GLCTNFTISRAILSD	0.767	12.49	1
13	ACO55067.2	LCTNFTISRAILSDA	0.788	9.87	0.5
14	ACO55067.2	CTNFTISRAILSDAV	0.792	9.45	0.4
15	ACO55067.2	TNFTISRAILSDAVA	0.792	9.53	0.4
16	ACO55067.2	NFTISRAILSDAVAL	0.77	12.08	0.9
17	ACO55067.2	LHRYSYKLAGVNQVD	0.78	10.82	0.7
18	ACO55067.2	HRYSYKLAGVNQVDV	0.803	8.43	0.25
19	ACO55067.2	RYSYKLAGVNQVDVV	0.812	7.68	0.17
20	ACO55067.2	YSYKLAGVNQVDVVR	0.789	9.78	0.5
21	ACO55067.2	DIGKSFELNQAARAV	0.792	9.53	0.4
22	ACO55067.2	IGKSFELNQAARAVT	0.805	8.23	0.25
23	ACO55067.2	GKSFELNQAARAVTQ	0.805	8.23	0.25
24	ACO55067.2	KSFELNQAARAVTQQ	0.798	8.93	0.3
25	ACO55067.2	AKTGFIANLVNSLHR	0.788	9.94	0.5
26	ACO55067.2	KTGFIANLVNSLHRH	0.793	9.42	0.4
27	ACO55067.2	TGFIANLVNSLHRHD	0.774	11.5	0.8

**Table 4:** NetMHCIIpan 3.2 scores for HLA-DRB01_1501

S. no.	Protein GenBank_ID	Epitope	DRB1_1501		
			1-log50k	nM	Rank
1	ACO55067.2	GVVLIMFNRFHNYVV	0.66	39.47	0.8
2	ACO55067.2	VVLIMFNRFHNYVVE	0.661	39.34	0.7
3	ACO55067.2	VLIMFNRFHNYVVEK	0.667	36.78	0.6
4	ACO55067.2	LIMFNRFHNYVVEKL	0.66	39.6	0.8
5	ACO55067.2	IMFNRFHNYVVEKLA	0.649	44.78	1
6	ACO55067.2	VFYKLVLRAFPNHFR	0.703	24.78	0.15
7	ACO55067.2	FYKLVLRAFPNHFRG	0.715	21.86	0.08
8	ACO55067.2	YKLVLRAFPNHFRGD	0.697	26.39	0.2
9	ACO55067.2	KLVLRAFPNHFRGDS	0.682	31.29	0.4
10	ACO55067.2	ITLKLLHRYSYKLAG	0.651	43.63	1
11	ACO55067.2	TLKLLHRYSYKLAGV	0.659	40.07	0.8
12	ACO55067.2	LKLLHRYSYKLAGVN	0.649	44.65	1

**Table 5:** NetMHCIIpan 3.2 scores for HLA-DRB01_1101

S. no.	Protein GenBank_ID	Epitope	DRB1_1101		
			1-log50k	nM	Rank
1	ACO55067.2	THVFYKLVLRAFPNH	0.667	36.66	1.3
2	ACO55067.2	HVFYKLVLRAFPNHF	0.673	34.51	1.2
3	ACO55067.2	VFYKLVLRAFPNHFR	0.689	29.05	0.8
4	ACO55067.2	FYKLVLRAFPNHFRG	0.69	28.52	0.8
5	ACO55067.2	LLLRYFMEGARIRSS	0.668	36.44	1.3
6	ACO55067.2	LLRYFMEGARIRSSV	0.676	33.27	1.1
7	ACO55067.2	LRYFMEGARIRSSVA	0.671	34.98	1.2
8	ACO55067.2	GARIRSSVALPRVVA	0.663	38.35	1.4
9	ACO55067.2	LTTMLKVIGRLDNLR	0.663	38.15	1.4
10	ACO55067.2	TTMLKVIGRLDNLRR	0.669	35.96	1.3
11	ACO55067.2	TMLKVIGRLDNLRRA	0.665	37.69	1.4

VaxiJen ver.2.0 tool was used to determine antigenicity of selected epitopes with threshold of 0.4 (Table 6). Antigenic epitopes were used for further physiochemical screening of epitopes.

**Table 6:** Epitope screening based on antigenicity scores (threshold value ≥ 0.4)

HLA determinant	Epitope	GenBank_ID	VaxiJen score	Antigenicity
DRB1_0101	VPERKFYLSAAPQCI	pdb_2XVN_B	0.461	Antigen
	LHRYSYKLAGVNQVD	ACO55067.2	0.554	Antigen
	HRYSYKLAGVNQVDV	ACO55067.2	0.834	Antigen
	RYSYKLAGVNQVDVV	ACO55067.2	0.559	Antigen
	GKSFELNQAARAVTQ	ACO55067.2	0.632	Antigen
	KSFELNQAARAVTQQ	ACO55067.2	0.485	Antigen
DRB1_1501	VFYKLVLRAFPNHFR	ACO55067.2	0.482	Antigen
	YKLVLRAFPNHFRGD	ACO55067.2	0.586	Antigen
	KLVLRAFPNHFRGDS	ACO55067.2	0.603	Antigen
	ITLKLLHRYSYKLAG	ACO55067.2	1.034	Antigen
	TLKLLHRYSYKLAGV	ACO55067.2	0.599	Antigen
	LKLLHRYSYKLAGVN	ACO55067.2	0.878	Antigen
DRB1_1101	HVFYKLVLRAFPNHF	ACO55067.2	0.511	Antigen
	VFYKLVLRAFPNHFR	ACO55067.2	0.482	Antigen
	LLLRYFMEGARIRSS	ACO55067.2	0.868	Antigen
	GARIRSSVALPRVVA	ACO55067.2	0.582	Antigen

### Physiochemical analysis of epitopes

Screened antigenic proteins were further analysed for physiochemical properties to screen epitopes on the basis of stability, half-life, isoelectric point, and GRAVY score (grand average of hydropathicity) (Table 7). Instability index defines protein structure to be unstable if greater than 50%, and half-life was calculated as per action data against mammalian reticulocytes by deploying ProtParam server of ExPASy tools. Similar epitopes with single or dual amino acid change were also removed from screened data, which finalizes 5 epitopes for further structural and docking analysis.

**Table 7:** Epitopes further screening based on physiochemical properties

Epitope	Mol. wt.	Isoelectric point	Half-life	Instability index	GRAVY score	Inference
VPERKFYLSAAPQCI	1722	8.74	100 h	76.5 (unstable)	0.047	Not selected
LHRYSYKLAGVNQVD	1762	8.5	5.5 h	−21.43 (stable)	−0.54	Selected
RYSYKLAGVNQVDVV	1710	8.5	1 h	−21.43 (stable)	−0.020	Selected
GKSFELNQAARAVTQ	1619	8.75	30 h	−12.04 (stable)	−0.54	Selected
VFYKLVLRAFPNHFR	1907	11	100 h	44.17 (partially stable)	0.247	Not selected
KLVLRAFPNHFRGDS	1757	10.84	3 h	29.62 (stable)	−0.447	Selected
ITLKLLHRYSYKLAG	1776	10	20 h	−12.67(stable)	0.1	Selected
HVFYKLVLRAFPNHF	1888	9.99	3.5 h	44.17 (partially stable)	0.333	Not selected
LLLRYFMEGARIRSS	1812	10.74	5.5 h	53.62 (unstable)	0.14	Not selected
GARIRSSVALPRVVA	1551	12.3	30 h	53.62 (unstable)	0.613	Not selected

### Structure prediction for selected epitopes

**Fig. 2:**
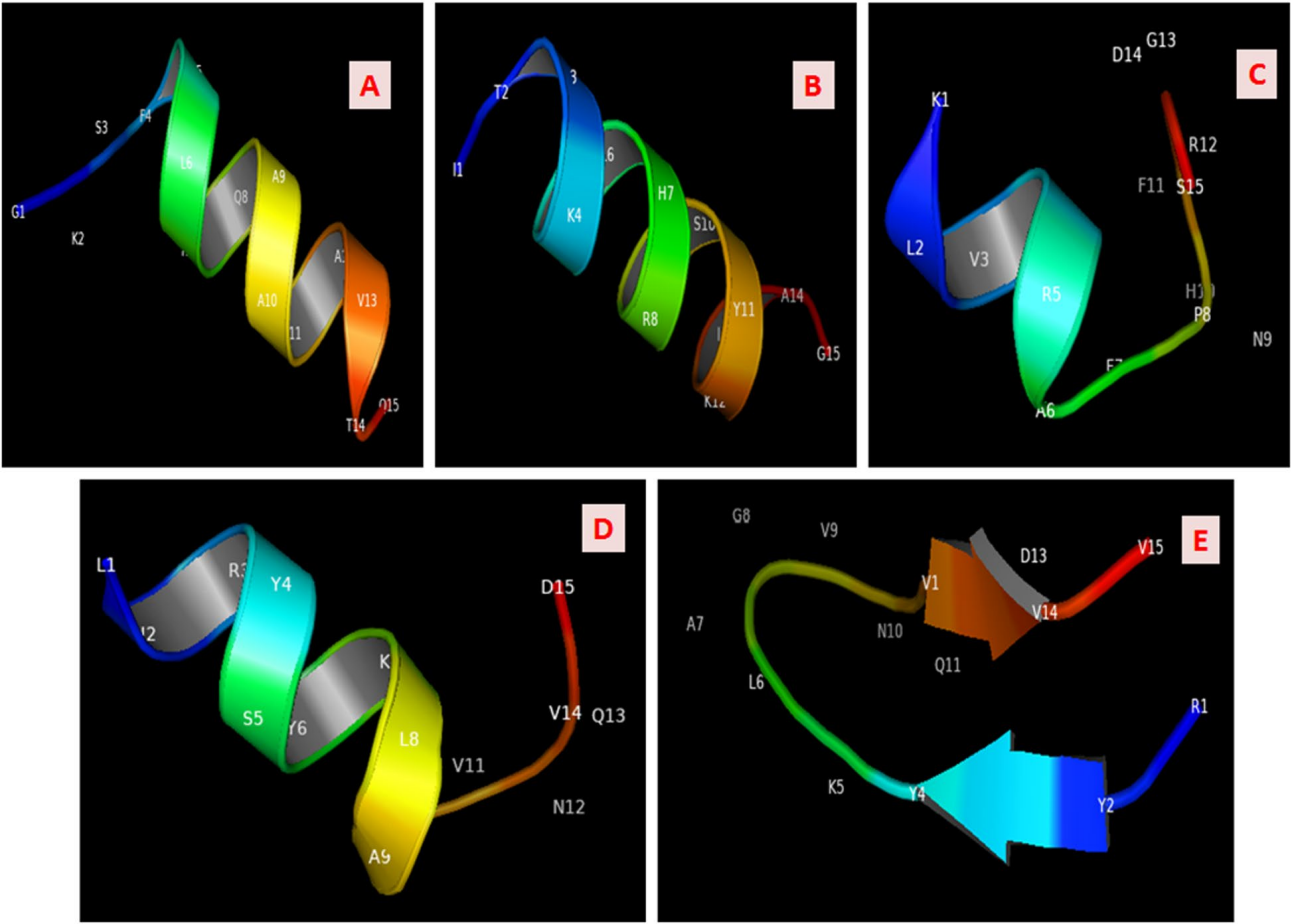
Epitopes 3D structures. **A** GKSFELNQAARAVTQ. **B** ITLKLLHRYSYKLAG. **C** KLVLRAFPNHFRGDS. **D** LHRYSYKLAGVNQVD. **E** RYSYKLAGVNQVDVV

Structural alphabet approach of de novo prediction was deployed to model the finalized epitopes structures. The PEP-FOLD ver.3.5 tool uses 5 to 50 amino residues for structure modelling and also performs 100 short simulations before conformation finalization for the provided sequence data, as this tool uses machine learning algorithms. Structures of epitopes modelled (Fig. 2) were used for further molecular docking studies with known crystal structures of HLA-allelic determinants that were downloaded from RCSB-PDB database; for HLA DRB01_0101 retrieval, PDB_ID is 1AQD, and for HLA DRB01_1501 retrieval, PDB_ID is 1XR9.

### Molecular docking

**Fig. 3:**
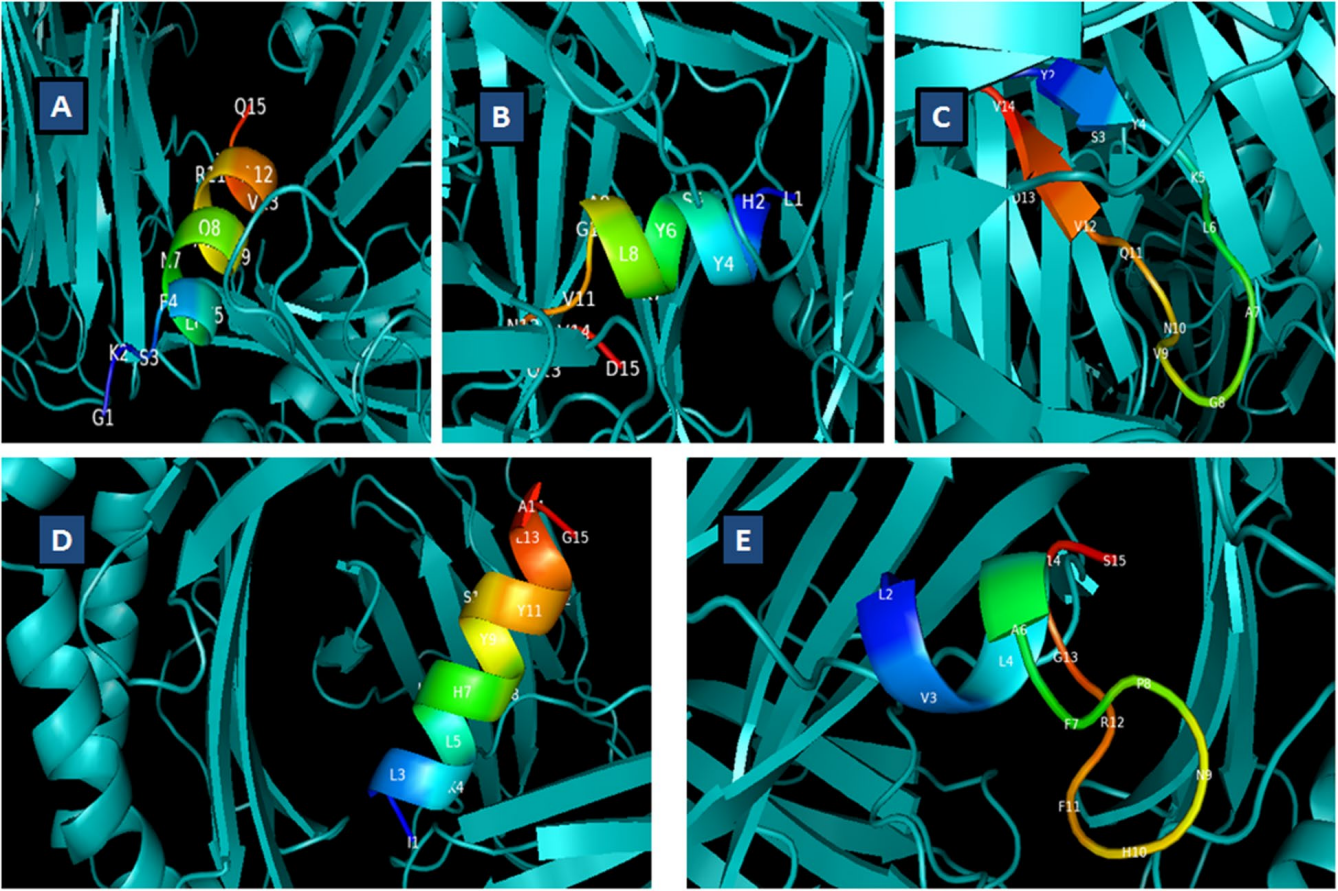
Docked complexes of epitopes with HLA-allelic determinants. **A** 1AQD-GKSFELNQAARAVTQ. **B** 1AQD-LHRYSYKLAGVNQVD. **C** 1AQD-RYSYKLAGVNQVDVV. **D** 1XR9-ITLKLLHRYSYKLAG. **E** 1XR9-KLVLRAFPNHFRGDS

HLA alleles HLA-DRB01:0101 and HLA-DRB01:15:01 were docked with epitopes that show interaction as per NetMHCIIpan 3.2 scores and previous screening in present research context. For molecular docking, PatchDock and FireDock tools were used. The atomic contact energy (ACE value) for docked complexes was provided in Table 8. ACE value for GKSFELNQAARAVTQ, ITLKLLHRYSYKLAG, KLVLRAFPNHFRGDS, LHRYSYKLAGVNQVD, and RYSYKLAGVNQVDVV epitopes show values less than −5 Kcal/mol in docked state with HLA allelic determinants. In Fig. 3, all the 5 docked complexes were shown that reveals fine interactions between receptor and ligand (epitopes).

**Table 8:** Molecular docking analysis: receptor and ligand docking scores

HLA-allelic determinant (receptor)	Epitope of interest (Ligand)	Atomic contact energy (Kcal/mol)
1XR9	ITLKLLHRYSYKLAG	−6.903
1XR9	KLVLRAFPNHFRGDS	−6.405
1AQD	RYSYKLAGVNQVDVV	−5.525
1AQD	GKSFELNQAARAVTQ	−5.452
1AQD	LHRYSYKLAGVNQVD	−6.325

### Docked complexes structural validation

**Fig. 4:**
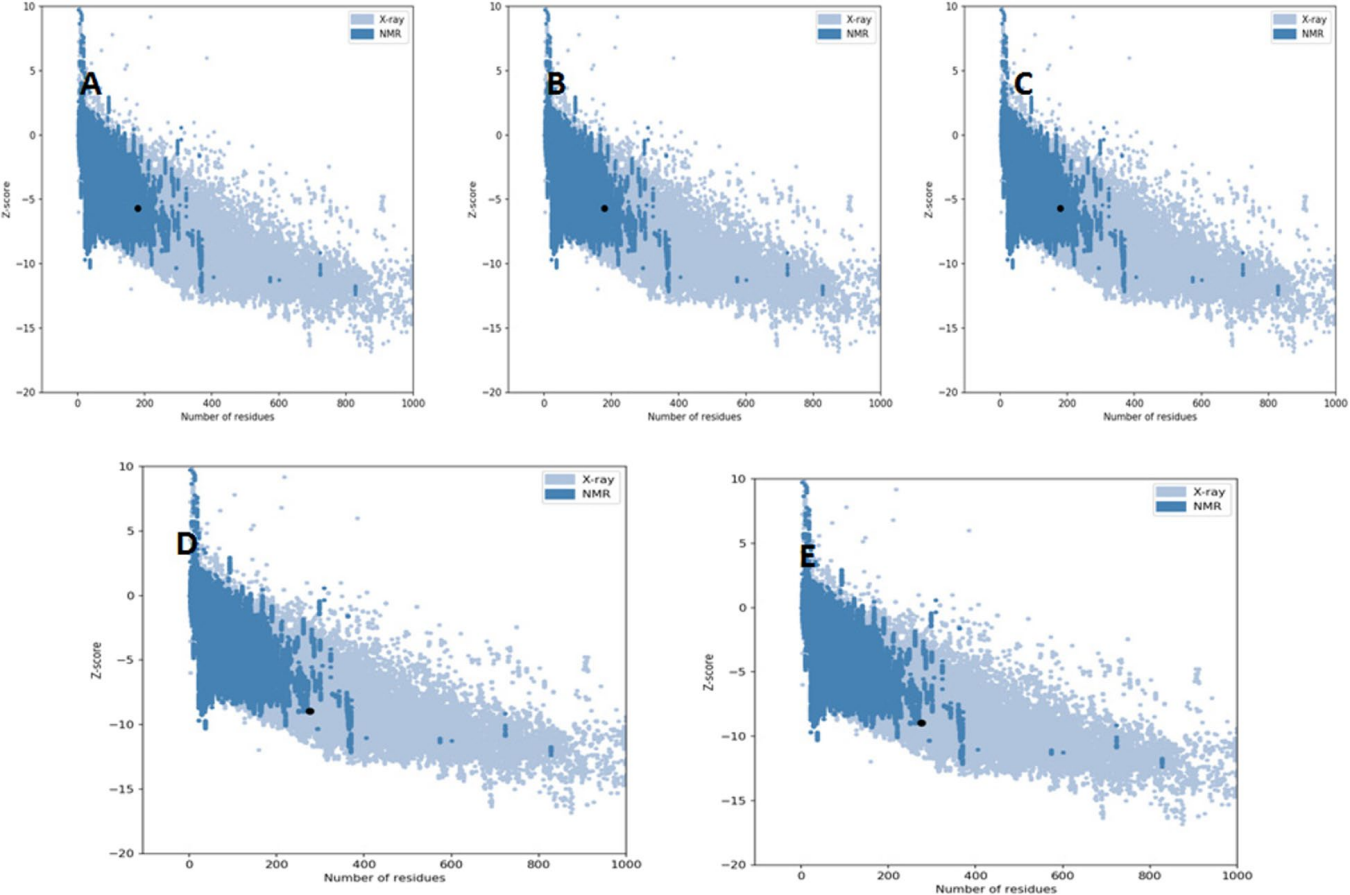
Z-plots for all the docked complex structures. **A** 1AQD-GKSFELNQAARAVTQ. **B** 1AQD-LHRYSYKLAGVNQVD. **C** 1AQD-RYSYKLAGVNQVDVV. **D** 1XR9-ITLKLLHRYSYKLAG. **E** 1XR9-KLVLRAFPNHFRGDS

Z-score indicates stability of structure and overall quality of the structure modelled with available datasets of X-ray and NMR models. The calculated Z-scores for complexes are as follows: (1) 1AQD-GKSFELNQAARAVTQ: −5.71; (2) 1AQD-LHRYSYKLAGVNQVD: −5.72; (3) 1AQD-RYSYKLAGVNQVDVV: −5.71; (4) 1XR9-ITLKLLHRYSYKLAG: −8.96; and (5) 1XR9-KLVLRAFPNHFRGDS: −8.96. Figure 4 indicates Z-plots for all the docked complex structures.

### Ramachandran plot analysis of docked complexes

**Fig. 5:**
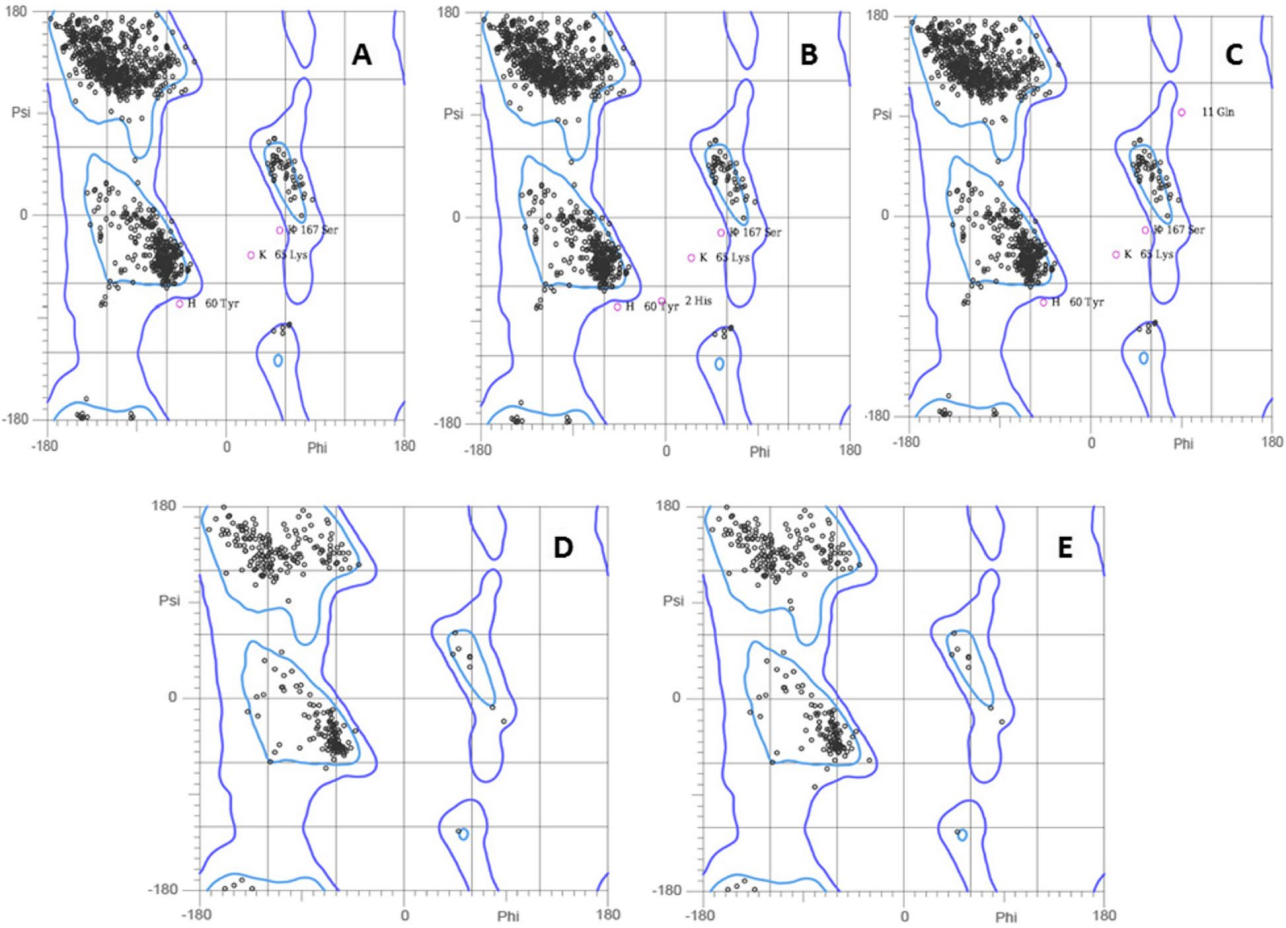
Ramachandran plots for docked complexes. **A** 1AQD-GKSFELNQAARAVTQ. **B** 1AQD-LHRYSYKLAGVNQVD. **C** 1AQD-RYSYKLAGVNQVDVV. **D** 1XR9-ITLKLLHRYSYKLAG. **E** 1XR9-KLVLRAFPNHFRGDS

MolProbity tool was deployed to reveal the validation of secondary structures of docked complexes by generating Ramachandran plots (Fig. 5), and it was noted that all the residues (above 90%) were found to be in favourable region or allowed region.

### MD-simulation analysis

**Fig. 6:**
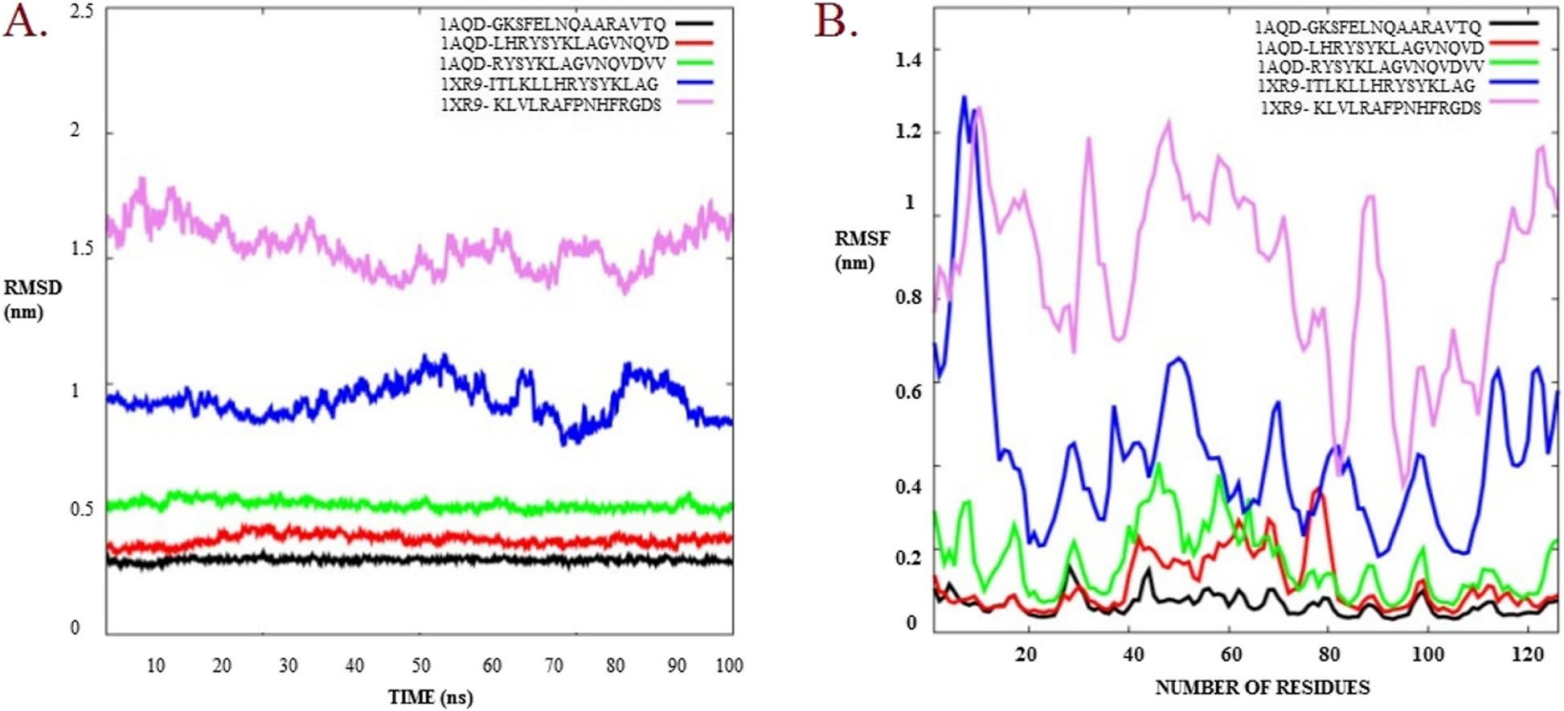
RMSD (**A**) and RMSF (**B**) plots for all the five docked complexes

The experimental characteristics of the dimension of sustainability and thermodynamics stages were reproduced using this force field for 100 ns. Moreover, within those sorts of simulations, where we examine behaviour at temperatures beyond 300 K, the choice of the water model is critical. TIP4P, a four-water system, was identified as the fine water model for this research. Here docked complexes were analysed for good interaction studies. An RMSD and RMSF plot clearly indicates that all the complexes hold values under 0 to 2.5 nm and 0 to 1.4 nm respectively, as provided in Fig. 6. Such scores indicate stability of complexes under longer durations.

## Discussion

Among the most common causes of invasive aspergillosis and acute bronchopulmonary aspergillosis is *Aspergillus fumigatus* [2]. Transmission with *A. fumigatus* produces aggressive aspergillosis in allogeneic hematopoietic stem cell transplant recipients, HIV patients, and cancer patients. Asthmatics and cystic fibrosis patients are allergic to *A. fumigatus* [3, 4]. The majority of T cells might belong to one of two subsets, which are attributed to the presence of one of two glycoproteins on their surface, labelled as CD8 or CD4. CD4 T cells serve as T-helper (Th) cells, recognizing peptides on MHC-II determinants [20]. The immune system’s hierarchical and combinatorial features contribute to its complexity. As a result, a massive quantity of data about immune systems is being created. This intricacy must be addressed in immunologic research. In current research, we found multiple epitopes: ITLKLLHRYSYKLAG, KLVLRAFPNHFRGDS, RYSYKLAGVNQVDVV, GKSFELNQAARAVTQ, and LHRYSYKLAGVNQVD from crucial proteins of *A. fumigatus* 5,8-linoleate diol synthase (ACO55067.2). ChainB-chitinase A1 (2XVN_B), RYSYKLAGVNQVDVV, GKSFELNQAARAVTQ, and LHRYSYKLAGVNQVD epitopes interact with HLA-DRB01_0101, while ITLKLLHRYSYKLAG and KLVLRAFPNHFRGDS epitopes interact with HLA-DRB01_1501. Molecular docking analysis reveals atomic contact energy (ACE) value for these five epitopes shown below −5 Kcal/mol in docked state. Also, docked complex was analysed for simulation analysis, and it was found that they show stable interaction pattern as per the RMSD and RMSF plots. Many previous studies show the importance of immunoinformatic study to support our analysis on fungal epitope determination likewise for *Candida auris*, *Tropheryma whipplei* [21, 22], dengue [23], human cytomegalovirus [24], and chikungunya [25]. Modern chemi-informatic and immunoinformatics study not only supports rapid vaccine prediction but also provides efficient economic resource management [26–28], although immunoinformatic requires wet-lab support as future perspectives for epitope synthesis and animal cell line-dependent validations.

## Conclusions

The invasive aspergillosis and acute bronchopulmonary aspergillosis are caused by harmful fungal pathogen *Aspergillus fumigatus*. Our modern immunoinformatic research shows ITLKLLHRYSYKLAG, KLVLRAFPNHFRGDS, RYSYKLAGVNQVDVV, GKSFELNQAARAVTQ, and LHRYSYKLAGVNQVD epitopes could bind to MHC-II HLA allelic determinants and can initiate immunogenic response in patients affected by *Aspergillus fumigatus*.

## Data Availability

All data is provided in manuscript.

## Abbreviations

RMSD: Root-mean-square deviation
RMSF: Root-mean-square fluctuation
MD: Molecular dynamics
ACE: Atomic contact energy
